# Poly[aqua­[μ_3_-5-(2-carboxyl­atophen­yl)-1*H*-tetra­zolato]zinc(II)]

**DOI:** 10.1107/S1600536808013688

**Published:** 2008-05-14

**Authors:** Xiu-Zhi Li, Zhi-Rong Qu

**Affiliations:** a Ordered Matter Science Research Center, College of Chemistry and Chemical Engineering, Southeast University, Nanjing 210096, People’s Republic of China

## Abstract

The title coordination polymer, [Zn(C_8_H_4_N_4_O_2_)(H_2_O)]_*n*_, was prepared by the hydro­thermal reaction of zinc nitrate and 2-(1*H*-tetra­zol-5-yl)benzoic acid. Two types of coordinated zinc cations exist in the structure. One is tetra­hedrally coordinated by two O and two N from two ligands, the other is octa­hedrally coordinated by two N and two O from two ligands at equatorial sites and by two O atoms of water mol­ecules at axial sites, resulting in a two-dimensional framework. The crystal structure is stabilized by intra­molecular O—H⋯O and O—H⋯N hydrogen bonds.

## Related literature

For the chemistry of tetra­zoles, see: Xiong *et al.* (2002 ▶); Xue *et al*. (2002 ▶); Dunica *et al.* (1991 ▶); Wang *et al.* (2005 ▶); Wittenberger *et al.* (1993 ▶); Hu *et al.* (2007 ▶). For the crystal structure of a related compound, see: Li *et al.* (2005 ▶).
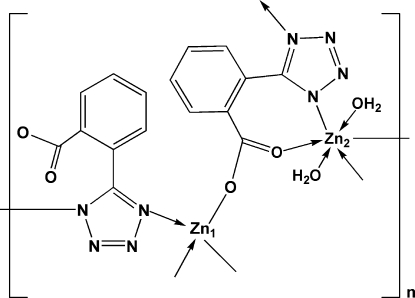


## Experimental

### 

#### Crystal data

[Zn(C_8_H_4_N_4_O_2_)(H_2_O)]
*M* *_r_* = 271.56
Monoclinic, 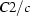

*a* = 19.696 (8) Å
*b* = 7.1340 (18) Å
*c* = 14.932 (6) Å
β = 114.39 (2)°
*V* = 1910.9 (12) Å^3^
*Z* = 8
Mo *K*α radiation
μ = 2.57 mm^−1^
*T* = 293 (2) K
0.07 × 0.07 × 0.06 mm

#### Data collection

Rigaku SCXmini diffractometer
Absorption correction: multi-scan (*CrystalClear*; Rigaku, 2005 ▶) *T*_min_ = 0.835, *T*_max_ = 0.860
9320 measured reflections
2173 independent reflections
1623 reflections with *I* > 2σ(*I*)
*R*_int_ = 0.085

#### Refinement

*R*[*F* ^2^ > 2σ(*F* ^2^)] = 0.050
*wR*(*F* ^2^) = 0.112
*S* = 1.08
2173 reflections
147 parameters
H-atom parameters constrained
Δρ_max_ = 0.63 e Å^−3^
Δρ_min_ = −0.60 e Å^−3^

### 

Data collection: *CrystalClear* (Rigaku, 2005 ▶); cell refinement: *CrystalClear*; data reduction: *CrystalClear*; program(s) used to solve structure: *SHELXS97* (Sheldrick, 2008 ▶); program(s) used to refine structure: *SHELXL97* (Sheldrick, 2008 ▶); molecular graphics: *SHELXTL* (Sheldrick, 2008 ▶); software used to prepare material for publication: *PRPKAPPA* (Ferguson, 1999 ▶).

## Supplementary Material

Crystal structure: contains datablocks I, global. DOI: 10.1107/S1600536808013688/rz2210sup1.cif

Structure factors: contains datablocks I. DOI: 10.1107/S1600536808013688/rz2210Isup2.hkl

Additional supplementary materials:  crystallographic information; 3D view; checkCIF report

## Figures and Tables

**Table 1:** Hydrogen-bond geometry (Å, °)

*D*—H⋯*A*	*D*—H	H⋯*A*	*D*⋯*A*	*D*—H⋯*A*
O1*W*—H1*W*⋯O1^i^	0.82	2.08	2.804 (4)	147
O1*W*—H2*W*⋯N3^ii^	0.79	2.25	2.976 (5)	155

Symmetry codes: (i) 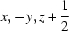; (ii) 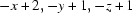.

**Fig. 1.**
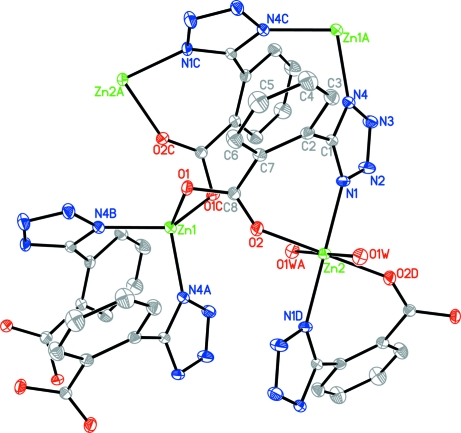
A partial packing diagram of the title compound, with displacement ellipsoids drawn at the 30% probability level. H atoms are omitted for clarity. [Symmetry codes: (A) 2-x, -y, 1-z; (B) x, y-1, z; (C) 2-x, y-1, 1/2-z; (D) 2-x, y, 1/2-z].

**Fig. 2.**
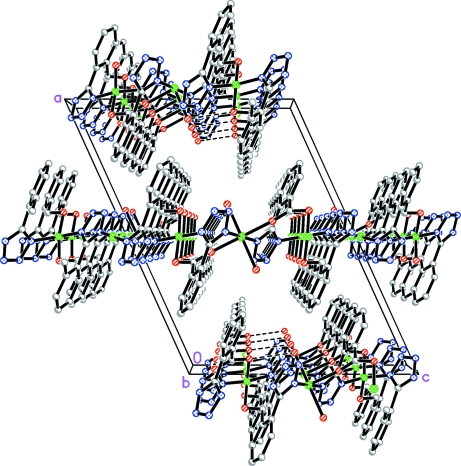
Packing diagram of the title compound, showing the structure along the b axis. H atoms are omitted for clarity.

## supplementary crystallographic information

### Comment

Coordination frameworks have received much attention over the past decade because of their potential applications. Multifunctional organic ligands are necessary to construct such frameworks. 2-(1*H*-Tetrazol-5-yl)benzoic acid is a ligand with two functional groups, a carboxylate group and a tetrazole ring. Tetrazole compounds have a wide range of applications in coordination chemistry, medicinal chemistry and material science (Hu, *et al.*, 2007; Xiong, *et al.*, 2002; Xue, *et al.*, 2002; Wang, *et al.*, 2005; Dunica, *et al.*, 1991; Wittenberger & Donner, 1993). We report here the crystal structure of the title compound, which was obtained by the hydrothermal reaction of zinc nitrate and 2-(1*H*-tetrazol-5-yl)benzoic acid.

In the structure of the title compound, two types of coordinated zinc cations exist (Fig. 1). Zn1 is tetrahedrally coordinated by two O and two N from two ligands, while Zn2 is octahedrally coordinated, with two N and two O from two ligands at equatorial sites and two O atoms of H_2_O molecules at axial sites, resulting in a two-dimensional framework (Fig 2). Bond lengths and angles in the compound are within normal ranges (Li *et al.*, 2005). The crystal structure is stabilized by intramolecular O—H···O and O—H···N hydrogen bonds (Table 1).

### Experimental

A mixture of Zn(NO_3_)_2_ (0.2 mmol) and 2-(1*H*-tetrazol-5-yl)benzoic acid (0.2 mmol) in H_2_O (4 ml) was heated in Pyrex tube at 100°C for two days. After slowly cooling down to room temperature over a period of 12 h, colourless crystals of the title compound suitable for diffraction were isolated.

### Refinement

Positional parameters of all the H atoms were calculated geometrically and were allowed to ride on their parent atoms, with C—H = 0.93 Å, O—H = 0.81Å and with U_iso_(H) = 1.2 U_eq_(C) or 1.5 U_eq_(O).

### Crystal data

**Table:**

[Zn(C_8_H_4_N_4_O_2_)(H_2_O)]	*F*_000_ = 1088.0
*M**_r_* = 271.56	*D*_x_ = 1.888 Mg m^−^^3^
Monoclinic, *C*2/*c*	Mo *K*α radiation λ = 0.71073 Å
Hall symbol: -C 2yc	Cell parameters from 1979 reflections
*a* = 19.696 (8) Å	θ = 3.1–27.5º
*b* = 7.1340 (18) Å	µ = 2.57 mm^−^^1^
*c* = 14.932 (6) Å	*T* = 293 (2) K
β = 114.39 (2)º	Block, colourless
*V* = 1910.9 (12) Å^3^	0.07 × 0.07 × 0.06 mm
*Z* = 8	

### Data collection

**Table:**

Rigaku SCXmini diffractometer	2173 independent reflections
Radiation source: fine-focus sealed tube	1623 reflections with *I* > 2σ(*I*)
Monochromator: graphite	*R*_int_ = 0.085
Detector resolution: 13.6612 pixels mm^-1^	θ_max_ = 27.5º
*T* = 293(2) K	θ_min_ = 3.1º
ω scans	*h* = −25→25
Absorption correction: multi-scan(CrystalClear; Rigaku, 2005)	*k* = −9→9
*T*_min_ = 0.835, *T*_max_ = 0.860	*l* = −19→19
9320 measured reflections	

### Refinement

**Table:**

Refinement on *F*^2^	Secondary atom site location: difference Fourier map
Least-squares matrix: full	Hydrogen site location: inferred from neighbouring sites
*R*[*F*^2^ > 2σ(*F*^2^)] = 0.050	H-atom parameters constrained
*wR*(*F*^2^) = 0.112	*w* = 1/[σ^2^(*F*_o_^2^) + (0.0929*P*)^2^ + 0.0509*P*] where*P* = (*F*_o_^2^ + 2*F*_c_^2^)/3
*S* = 1.08	(Δ/σ)_max_ < 0.001
2173 reflections	Δρ_max_ = 0.63 e Å^−^^3^
147 parameters	Δρ_min_ = −0.60 e Å^−^^3^
Primary atom site location: structure-invariant direct methods	Extinction correction: none

### Special details

**Table:**

Geometry. All e.s.d.'s (except the e.s.d. in the dihedral angle between two l.s. planes) are estimated using the full covariance matrix. The cell e.s.d.'s are taken into account individually in the estimation of e.s.d.'s in distances, angles and torsion angles; correlations between e.s.d.'s in cell parameters are only used when they are defined by crystal symmetry. An approximate (isotropic) treatment of cell e.s.d.'s is used for estimating e.s.d.'s involving l.s. planes.
Refinement. Refinement of *F*^2^ against ALL reflections. The weighted *R*-factor *wR* and goodness of fit *S* are based on *F*^2^, conventional *R*-factors *R* are based on *F*, with *F* set to zero for negative *F*^2^. The threshold expression of *F*^2^ > σ(*F*^2^) is used only for calculating *R*-factors(gt) *etc*. and is not relevant to the choice of reflections for refinement. *R*-factors based on *F*^2^ are statistically about twice as large as those based on *F*, and *R*- factors based on ALL data will be even larger.

### Fractional atomic coordinates and isotropic or equivalent isotropic displacement parameters (Å^2^)

**Table:**

	*x*	*y*	*z*	*U*_iso_*/*U*_eq_	
Zn1	1.0000	−0.31787 (9)	0.2500	0.0245 (2)	
Zn2	1.0000	0.0000	0.5000	0.0242 (2)	
C1	0.9761 (2)	0.3503 (5)	0.3649 (3)	0.0202 (8)	
C2	0.8990 (2)	0.3170 (6)	0.2905 (3)	0.0213 (8)	
C3	0.8527 (2)	0.4731 (6)	0.2629 (3)	0.0289 (10)	
H3	0.8693	0.5859	0.2959	0.035*	
C4	0.7821 (3)	0.4630 (6)	0.1866 (4)	0.0383 (12)	
H4	0.7514	0.5681	0.1693	0.046*	
C5	0.7577 (3)	0.2978 (7)	0.1370 (4)	0.0410 (12)	
H5	0.7104	0.2910	0.0858	0.049*	
C6	0.8032 (3)	0.1407 (6)	0.1630 (3)	0.0339 (11)	
H6	0.7864	0.0299	0.1279	0.041*	
C7	0.8733 (2)	0.1459 (6)	0.2405 (3)	0.0222 (9)	
C8	0.9165 (2)	−0.0327 (5)	0.2671 (3)	0.0217 (9)	
N1	1.0151 (2)	0.2573 (5)	0.4466 (2)	0.0241 (8)	
N2	1.0788 (2)	0.3533 (5)	0.4939 (3)	0.0295 (9)	
N3	1.0792 (2)	0.4999 (5)	0.4424 (3)	0.0280 (8)	
N4	1.01491 (19)	0.5018 (4)	0.3598 (2)	0.0220 (7)	
O1	0.91662 (17)	−0.1326 (4)	0.1958 (2)	0.0270 (7)	
O2	0.94994 (17)	−0.0846 (4)	0.3536 (2)	0.0314 (7)	
O1W	0.89412 (16)	0.0990 (4)	0.4984 (2)	0.0322 (7)	
H1W	0.8913	0.0681	0.5497	0.048*	
H2W	0.8872	0.2061	0.5035	0.048*	

### Atomic displacement parameters (Å^2^)

**Table:**

	*U*^11^	*U*^22^	*U*^33^	*U*^12^	*U*^13^	*U*^23^
Zn1	0.0335 (4)	0.0167 (3)	0.0215 (4)	0.000	0.0097 (3)	0.000
Zn2	0.0341 (4)	0.0186 (4)	0.0186 (4)	−0.0009 (3)	0.0097 (3)	0.0027 (3)
C1	0.026 (2)	0.0153 (19)	0.021 (2)	−0.0018 (17)	0.0104 (17)	0.0000 (16)
C2	0.021 (2)	0.022 (2)	0.0183 (19)	−0.0016 (17)	0.0057 (17)	0.0025 (16)
C3	0.032 (2)	0.023 (2)	0.030 (2)	0.0042 (19)	0.011 (2)	0.0001 (18)
C4	0.031 (3)	0.033 (3)	0.045 (3)	0.013 (2)	0.009 (2)	0.003 (2)
C5	0.026 (2)	0.043 (3)	0.039 (3)	0.004 (2)	−0.002 (2)	−0.001 (2)
C6	0.029 (2)	0.029 (2)	0.035 (3)	0.001 (2)	0.004 (2)	−0.004 (2)
C7	0.024 (2)	0.019 (2)	0.022 (2)	0.0019 (17)	0.0094 (18)	0.0021 (16)
C8	0.023 (2)	0.021 (2)	0.021 (2)	−0.0050 (16)	0.0088 (18)	−0.0013 (16)
N1	0.032 (2)	0.0186 (17)	0.0189 (17)	−0.0044 (15)	0.0083 (16)	0.0033 (14)
N2	0.029 (2)	0.0235 (19)	0.030 (2)	−0.0030 (16)	0.0062 (17)	0.0079 (16)
N3	0.031 (2)	0.0223 (19)	0.0235 (19)	−0.0053 (15)	0.0041 (17)	0.0025 (14)
N4	0.0271 (18)	0.0168 (17)	0.0210 (18)	−0.0020 (14)	0.0088 (15)	0.0001 (13)
O1	0.0390 (18)	0.0226 (15)	0.0179 (14)	0.0056 (13)	0.0101 (13)	−0.0029 (12)
O2	0.048 (2)	0.0232 (16)	0.0191 (15)	0.0050 (14)	0.0100 (15)	−0.0004 (12)
O1W	0.0385 (18)	0.0309 (17)	0.0284 (17)	0.0003 (15)	0.0150 (15)	−0.0012 (14)

### Geometric parameters (Å, °)

**Table:**

Zn1—O1^i^	2.000 (3)	C3—H3	0.9300
Zn1—O1	2.000 (3)	C4—C5	1.369 (6)
Zn1—N4^ii^	2.008 (3)	C4—H4	0.9300
Zn1—N4^iii^	2.008 (3)	C5—C6	1.387 (6)
Zn2—N1	2.072 (3)	C5—H5	0.9300
Zn2—N1^iv^	2.072 (3)	C6—C7	1.389 (6)
Zn2—O2^iv^	2.082 (3)	C6—H6	0.9300
Zn2—O2	2.082 (3)	C7—C8	1.491 (5)
Zn2—O1W^iv^	2.193 (3)	C8—O2	1.240 (5)
Zn2—O1W	2.193 (3)	C8—O1	1.281 (5)
C1—N1	1.321 (5)	N1—N2	1.345 (5)
C1—N4	1.343 (5)	N2—N3	1.300 (4)
C1—C2	1.484 (5)	N3—N4	1.353 (5)
C2—C3	1.390 (5)	N4—Zn1^v^	2.008 (3)
C2—C7	1.411 (5)	O1W—H1W	0.8200
C3—C4	1.389 (6)	O1W—H2W	0.7851
			
O1^i^—Zn1—O1	97.27 (17)	C2—C3—H3	119.5
O1^i^—Zn1—N4^ii^	124.90 (13)	C5—C4—C3	119.8 (4)
O1—Zn1—N4^ii^	105.93 (12)	C5—C4—H4	120.1
O1^i^—Zn1—N4^iii^	105.93 (12)	C3—C4—H4	120.1
O1—Zn1—N4^iii^	124.90 (13)	C4—C5—C6	120.2 (4)
N4^ii^—Zn1—N4^iii^	100.34 (19)	C4—C5—H5	119.9
N1—Zn2—N1^iv^	180.000 (1)	C6—C5—H5	119.9
N1—Zn2—O2^iv^	93.81 (13)	C5—C6—C7	121.1 (4)
N1^iv^—Zn2—O2^iv^	86.19 (13)	C5—C6—H6	119.4
N1—Zn2—O2	86.19 (13)	C7—C6—H6	119.4
N1^iv^—Zn2—O2	93.81 (13)	C6—C7—C2	118.7 (4)
O2^iv^—Zn2—O2	180.000 (1)	C6—C7—C8	117.4 (4)
N1—Zn2—O1W^iv^	90.11 (13)	C2—C7—C8	123.9 (3)
N1^iv^—Zn2—O1W^iv^	89.89 (13)	O2—C8—O1	121.1 (4)
O2^iv^—Zn2—O1W^iv^	92.66 (12)	O2—C8—C7	122.1 (4)
O2—Zn2—O1W^iv^	87.34 (12)	O1—C8—C7	116.8 (4)
N1—Zn2—O1W	89.89 (13)	C1—N1—N2	106.9 (3)
N1^iv^—Zn2—O1W	90.11 (13)	C1—N1—Zn2	132.8 (3)
O2^iv^—Zn2—O1W	87.34 (12)	N2—N1—Zn2	120.0 (3)
O2—Zn2—O1W	92.66 (12)	N3—N2—N1	109.3 (3)
O1W^iv^—Zn2—O1W	180.000 (1)	N2—N3—N4	108.4 (3)
N1—C1—N4	109.2 (4)	C1—N4—N3	106.3 (3)
N1—C1—C2	129.5 (4)	C1—N4—Zn1^v^	131.8 (3)
N4—C1—C2	121.1 (3)	N3—N4—Zn1^v^	121.1 (3)
C3—C2—C7	119.2 (4)	C8—O1—Zn1	108.4 (3)
C3—C2—C1	115.9 (4)	C8—O2—Zn2	145.6 (3)
C7—C2—C1	124.5 (4)	Zn2—O1W—H1W	109.5
C4—C3—C2	120.9 (4)	Zn2—O1W—H2W	121.0
C4—C3—H3	119.5	H1W—O1W—H2W	95.2

Symmetry codes: (i) −*x*+2, *y*, −*z*+1/2; (ii) −*x*+2, *y*−1, −*z*+1/2; (iii) *x*, *y*−1, *z*; (iv) −*x*+2, −*y*, −*z*+1; (v) *x*, *y*+1, *z*.

### Hydrogen-bond geometry (Å, °)

**Table:**

*D*—H···*A*	*D*—H	H···*A*	*D*···*A*	*D*—H···*A*
O1W—H1W···O1^vi^	0.82	2.08	2.804 (4)	147
O1W—H2W···N3^vii^	0.79	2.25	2.976 (5)	155

Symmetry codes: (vi) *x*, −*y*, *z*+1/2; (vii) −*x*+2, −*y*+1, −*z*+1.
